# Introducing the thematic series on transcranial direct current stimulation (tDCS) for motor rehabilitation: on the way to optimal clinical use

**DOI:** 10.1186/s12984-019-0507-y

**Published:** 2019-03-04

**Authors:** Rodrigo Vitório, Samuel Stuart, Leigh E. Charvet, Alan Godfrey

**Affiliations:** 1 São Paulo State University (Unesp), Institute of Biosciences, Posture and Gait Studies Laboratory (LEPLO), Rio Claro, Brazil; 2 Department of Neurology, Oregon Health & Science University, Portland, OR USA; 3 Department of Neurology, NYU School of Medicine, New York, NY USA; 4 Department of Computer and Information Science, Northumbria University, Newcastle upon Tyne, UK

## Introduction

Transcranial direct current stimulation (tDCS) is a method of noninvasive brain stimulation that directs a constant low amplitude electric current through scalp electrodes. tDCS has been shown to modulate excitability in both cortical and subcortical brain areas [1, 2], with anodal tDCS leading to increased neuronal excitability and cathodal tDCS inversely leading to reduced neuronal excitability. tDCS can also modulate blood flow (i.e. oxygen supply to cortical and subcortical areas [3]) and neuronal synapsis strength [4], triggering plasticity processes (i.e. long-term potentiation and long-term depression). There is growing interest in using tDCS as a low-cost, non-invasive brain stimulation option for a wide range of potential clinical applications. Advantages of tDCS over other methods of non-invasive brain stimulation include favorable safety and tolerability profiles and its portability and applicability.

The use of tDCS in motor rehabilitation for neurological diseases as well as in healthy ageing is a growing area of therapeutic use. Although the results of tDCS interventions for motor rehabilitation are still preliminary, they encourage further research to better understand its therapeutic utility and to inform optimal clinical use. Therefore, The Journal of NeuroEngineering and Rehabilitation (JNER. https://jneuroengrehab.biomedcentral.com/) is pleased to present the thematic series entitled “tDCS application for motor rehabilitation”.

The goal of this thematic series is to increase the awareness of academic and clinical communities to different potential applications of tDCS for motor rehabilitation. Experts in the field were invited to submit experimental or review studies. A call for papers was also announced to reach those interested in contributing to this thematic series. This collection of articles was thought to present the most recent advances in tDCS for motor rehabilitation, addressing topics such as theoretical, methodological, and practical approaches to be considered when designing tDCS-based rehabilitation. The targeted disorders include but are not limited to: stroke, Parkinson’s disease, Cerebral Palsy, cerebellar ataxia, trauma, Multiple Sclerosis.

## tDCS – A promising clinical tool for motor rehabilitation

tDCS has been used in experimental and clinical neuroscience for the study of brain functions and treatment in a range of disorders of the central nervous system. Of particular interest to this thematic series, a growing body of evidence suggest that tDCS has potential to become a clinical tool for motor rehabilitation.

The existing tDCS protocols using well-defined montages, stimulus durations and intensities are safe and well tolerated by both healthy individuals and clinical populations. There are no reported indications of any serious adverse effects, such as damage of brain tissue or seizure induction, with the use of 1–2 mA protocols [5–7]. The most commonly reported adverse effects included redness, tingling and itching sensations under the electrodes, as well as headache [6, 8]. Moreover, the overall adverse effect rates are similar between active and sham tDCS [6], which suggests that the mild adverse effects are related to electrode positioning on the skin and not the stimulation itself.

As tDCS is portable, devices can easily be transported, which circumvents accessibility barriers to health care (i.e. tDCS can easily be moved into clinics or wards). It can be implemented in combination with other kinds of interventions, such as cognitive or physical training or exercise, with this pairing possibly leading to synergistic benefit [9]. Although accumulating evidence highlights potential benefits offered by tDCS for motor rehabilitation, further research is required for tDCS to become an approved clinical tool. The majority of existing clinical trials has involved a limited number of participants, which may imply underpowered analysis. Thus, large-scale studies are needed to overcome this major flaw.

Due to the potential for self- or caregiver-application, remotely supervised protocols have been developed and recently found feasible for those with motor impairment [10]. However, these studies employ highly structured protocols and rigorous criteria with real time supervision via teleconference, and do not support a “do-it-yourself” tDCS practice. Instead, the remotely supervised protocols can be used to facilitate the clinical trial designs that are necessary in order to advance tDCS towards therapeutic use.

Data on optimal protocols and predictors of response to tDCS are currently lacking in the literature. Future studies in this field should focus on determining the optimal stimulation parameters and predictors of response to tDCS in different clinical populations. It seems that one size does not fit all in tDCS. However, previous studies may be limited, as standard clinical assessments may miss subtle motor improvements. Future outcomes for determining the effectiveness of tDCS for motor rehabilitation need to be robust. Therefore, combining tDCS protocols with other validated mobile technologies to monitor motor performance, such as wearable inertial sensors or innovative Internet of Things devices, may provide important insight into effectiveness within clinic and beyond.

Despite the positive progression of research to clinical practice, there are still questions to be answered before tDCS can be extensively recommended for motor rehabilitation.

• What is the ideal intensity and duration of the session?

• How many sessions are required?

• What is the ideal interval between sessions?

• What about patients’ characteristics?

• Who will benefit from tDCS?

• Do specific demographic characteristics lead to greater benefits?

## Final considerations

We hope the accepted papers will contribute meaningfully to the body of knowledge in the field of tDCS for motor rehabilitation and that they will motivate the development of further research. Additionally, we hope this thematic series will assist both researchers and clinical professionals in making decisions for the achievement of optimal benefits throughout tDCS.
